# Novel Therapeutic Effects of Leonurine On Ischemic Stroke: New Mechanisms of BBB Integrity

**DOI:** 10.1155/2017/7150376

**Published:** 2017-06-13

**Authors:** Qiu-Yan Zhang, Zhi-Jun Wang, De-Miao Sun, Ying Wang, Peng Xu, Wei-Jun Wu, Xin-Hua Liu, Yi-Zhun Zhu

**Affiliations:** ^1^Department of Pharmacology, School of Pharmacy and Institute of Biomedical Science, Fudan University, Shanghai, China; ^2^Department of Pharmacology, School of Pharmacy, Macau University of Science & Technology, Macau

## Abstract

Stroke is a leading cause of morbidity and mortality globally. Leonurine (also named SCM-198), a compound extracted from *Herba leonuri*, was effective on the prevention of various cardiovascular and brain diseases. The purpose of this study was to explore the possible therapeutic potential of SCM-198 against ischemia reperfusion injury and underlying mechanisms. In the in vivo transient middle cerebral artery occlusion (tMCAO) rat model, we found that treatment with SCM-198 could decrease infarct volume and improve neurological deficit by protecting against blood-brain barrier (BBB) breakdown. In the in vitro model of cell oxygen-glucose deprivation and reoxygenation (OGD/R), consistent results were obtained with decreased reactive oxygen species (ROS) production and maintained the BBB integrity. Further study demonstrated that SCM-198 increased the expression of histone deacetylase- (HDAC-) 4 which could inhibit NADPH oxidase- (NOX-) 4 and matrix metalloproteinase- (MMP-) 9 expression, resulting in the elevation of tight junction proteins, including claudin-5, occludin, and zonula occluden- (ZO-) 1. These results indicated SCM-198 protected BBB integrity by regulating the HDAC4/NOX4/MMP-9 tight junction pathway. Our findings provided novel insights into the protective effects and mechanisms of SCM-198 on ischemic stroke, indicating SCM-198 as a new class of potential drug against acute onset of ischemic stroke.

## 1. Introduction

Stroke is a main cause of morbidity and mortality throughout the world. In the clinic, stroke is divided into two forms: ischemic stroke which accounts for ~85% and hemorrhagic stroke, including intracerebral (~10%) and subarachnoidal (~3%) bleedings [1]. Ischemic stroke is mainly caused by blockage of the blood vessels, while hemorrhagic stroke by rupture of blood vessels. Currently, tissue-type plasminogen activator (tPA) serves as the priority remedy for ischemic stroke, from which only about 10% patients are suitable for this therapy. In addition, concerns have been raised regarding its limited therapeutic time window and safety issue, which result in less than 5% of clinical efficiency in patients [1, 2]. Meanwhile, the secondary damage caused by reperfusion will bring worse results, such as blood-brain barrier (BBB) breakdown, inflammation, and postischemic neuronal injury [1].

The BBB is composed of three main elements: brain microvessel endothelium, astrocyte endfeet, and pericytes, forming neurovascular unit (NVU). BBB was damaged in early critical event in ischemia-reperfusion (I/R) which causes the edema formation, inflammatory cascade, and ultimately serious outcomes [3]. BBB dysfunction is a peculiar character of many neurological conditions, for instance, ischemic and hemorrhagic stroke, multiple sclerosis, and brain tumours [4]. Currently, many researchers focus on neurons and brain parenchyma, whereas straight BBB protection has attracted little attention. The results show that early BBB disruption is the cause rather than the result of parenchymal cell injury [5]. Mounting evidences have suggested that brain microvessel endothelium cells (BMECs) are the basis of BBB which form a barrier that restricts diffusion of blood-borne solutes. BMECs play a central role in maintaining the BBB integrity by tight junctional proteins (TJs) and caveolin-1 (Cav1)—mediated trans-endothelia vesicular transport [6–9]. TJs contain occludin, claudins, junctional adhesion molecules (JAM), and cytoplasm accessory zonula occluden (ZO) proteins. Among them, occludin, claudin-5, and ZO-1 are widely investigated and considered as essential factors for BBB integrity. Occludin and claudin-5 are the transmembrane proteins which are responsible for fence function and paracellular size selectivity, respectively, while ZO-1 is in charge of anchoring them to the actin cytoskeleton [10]. Following subjected to transient middle cerebral artery occlusion (tMCAO) or oxygen-glucose deprivation and reoxygenation (OGD/R), loss of BMECs evoked stress fibre formation and TJ redistribution resulted in shrinked cell morphology, enhancing permeability of BBB. Targeting the BMEC structural changes could prevent BBB insult and secondary tissue injury [6]. As to Cav1, a recent study revealed that transcellular mechanisms mediated by Cav1 do not exert a predominant role up to 24 h after ischemia induced BBB breakdown [9]. So we chose bEnd.3 (brain microvascular endothelial cell line) in our experiment. And we determined the effect of SCM-198 on BBB integrity by TJs.

Recently, a lot of attention has been focused on Leonurine which also named SCM-198. As we know, SCM-198 is a unique single compound existed only in *Herba leonuri*, which is widely used in gynecology. Previous research suggested SCM-198 could improve antioxidant capacity of myocardium, promote angiogenesis in ischemic myocardium, and ameliorate endothelial dysfunction caused by hyperlipidemia [11, 12]. SCM-198 was surprisingly found to be effective in permanent MCAO stroke models via modulating mitochondrial functions [13], which prompted us to further explore its possible therapeutic potential in more dangerous tMCAO models. Therefore, we investigated the protective effects of SCM-198 in both tMCAO rat model and OGD/R cell model. Furthermore, we addressed new mechanisms that contribute to the protective effects of SCM-198 and whether they occur via maintaining BBB functioning and permeability.

## 2. Materials and Methods

### 2.1. Materials

Antibodies were obtained from the following commercial sources: Matrix metalloproteinase- (MMP-) 9 and histone deacetylase- (HDAC-) 4 (Cell Signaling Technology), claudin-5 and ZO-1 (Invitrogen), occludin, the nicotinamide adenine dinucleotide phosphate oxidase- (NOX-) 4, and GAPDH (Proteintech), and rhodamine phalloidin was obtained from Cytoskeleton. GKT137831 and Tasquinimod (Taq) was from selleck. Lactate dehydrogenase (LDH) activity assay kit was bought from Beyotime Institute of Biotechnology. Cell counting kit 8 (CCK8) was from Dojindo. 2,3,5-Triphenyltetrazolium chloride (TTC), 2′,7′-dichlorofluorescin diacetate (DCFH-DA), diphenyleneiodonium (DPI), fluorescein isothiocyanate- (FITC-) labeled dextran, and other chemical reagents were from Sigma-Aldrich.

### 2.2. Animal Treatment and Model

The experiment protocol was approved by the institutional ethical committee with internationally accepted ethical standards. All protocols and animal handling were performed in accordance under the guidelines of National Institutes of Health Guide for the Care and Use of Laboratory Animals. Hundred male Sprague-Dawley (SD) rats were supplied by the Laboratory Animal Center, Fudan University. Rats weighing 180–220 g were housed with food and water ad libitum under diurnal lighting condition. Rats were anaesthetized with chloral hydrate (300 mg/kg, intraperitoneally) as our standard protocol.

Briefly, we performed the surgery as described previously [14]. 90 minutes later, we withdrew the filament to cause the reperfusion injury. At different time point of MCAO and reperfusion, occlusion was confirmed by laser Doppler imaging system (Moor Instruments, USA) to monitor cerebral blood flow (CBF).

All the animals mentioned above were randomly divided into five groups: control operation group, tMCAO group treated with saline, vehicle group with Edaravone (3 mg/kg/day) for tMCAO [15], and treatment groups which were posttreated (0.5 h and 2 h after operation) with SCM-198 (15 mg/kg/day in normal saline). All the drugs were given intravenous injection once daily for 3 days.

### 2.3. Measurement of Infarct Volume and Brain Swelling after Ischemia-Reperfusion

The TTC staining assay is one of the most common methods to measure infarct volume which was measured as previously described [16]. At the third day after I/R, animals were anesthetized with chloral hydrate (300 mg/kg, i.p.) and decapitated. Rat brains were immediately removed and then dissected into 2 mm-thick coronal slices using a brain slice matrix. Then the slices were stained with TTC solution (1% TTC in PBS) at 37°C for 15 min then photographed. Infarct sizes were quantified using ImageJ software. The relative infarction volume percentage (RIVP) was calculated as following equation: RIVP = IVA/TA × 100%, where IVA and TA were infarcted area and total area of the coronal sections, respectively.

The cerebral edema volume was also measured. Edema volume = ([volume of ipsilateral hemisphere − volume of contralateral hemisphere]/volume of contralateral hemisphere) × 100% [17].

### 2.4. Behavioral Evaluation

To evaluate neuronal function impairment after ischemic stroke insult, a neurological deficit grading system with a scale of zero to 5 was carried out on all of the animals as described previously [18].

### 2.5. Brain Water Content

Three days after tMCAO, rats were anesthetized by 10% chloral hydrate. The brains were removed and separated into contralateral and ipsilateral hemisphere. The wet weight of ipsilateral hemisphere was recorded immediately. After dried at 50°C for 48 hours, the dry weight of these samples were obtained by an electronic analytical balance. The formula for calculating brain water content (BWC) was as follows: (wet weight − dry weight)/wet weight × 100% [19].

### 2.6. Evaluation of Blood-Brain Barrier Integrity

The effect of SCM-198 on BBB permeability was assessed by Evans blue (EB) test [20]. EB dye (80 mg/kg) in phosphate-buffered saline (PBS; pH 7.4) was injected into tail vein and allowed to circulate for 24 h. Then rats were anesthetized with chloral hydrate, and transcardially perfused with PBS, the brains were removed and divided into the right and left hemispheres. The left hemispheres were weighed and placed in 50% trichloroacetic acid solution to precipitate protein. The supernatant was diluted 4-fold with ethanol. The EB dye was measured by a microplate fluorescence reader (excitation 620 nm, emission 680 nm). We also measured the cerebral edema volume. Edema volume = ([volume of ipsilateral hemisphere − volume of contralateral hemisphere]/volume of contralateral hemisphere) × 100% [21].

### 2.7. bEnd.3 Cell Culture and Treatment

Mouse bEnd.3 cell was bought from American Type Culture Collection (ATCC). Cells were cultured with Dulbecco's modified Eagle medium (DMEM, Hyclone, USA), supplemented with 10% fetal bovine serum (FBS, Hyclone, USA) and 100 *μ*g/ml penicillin/streptomycin (Gibco), and cultured at 37°C containing 5% CO_2_ and 95% O_2_. All the cells used in the experiments were performed from passages 2 to 10.

To mimic ischemic-like conditions in vitro, bEnd.3 cells were exposed to OGD as we described previously [22]. In brief, the cells were washed with PBS three times then replaced with serum- and glucose-free medium (Invitrogen). The cells were placed in a Biospherix incubator chamber (ProOx C21, USA), which was flushed with 95% N_2_ and 5% CO_2_ for 6 h then transferred to 95% air, 5% CO_2_, and continued to be cultured in glucose-containing medium for 4 h each time. Control cells were cultured with norm-oxygenated and glucose-containing medium for the same period of time in normal condition. The cells were divided into five groups: control, OGD, and cells treated with SCM-198 (0.1 *μ*M, 1 *μ*M, and 10 *μ*M) 2 h before OGD. The inhibitors were added 1 h before OGD until the end of the experiment.

### 2.8. Cellular Viability

Cell viability was determined using the CCK8 assay in accordance with the manufacturer's instructions [23]. In briefly, cells were seeded into 96-well plate at a density of 1.0 × 10^4^ cells/well and cultured for 24 h, and then 10 *μ*l CCK-8 solution was added to each well after reperfusion and incubated for 1 h. The absorbance was measured at 450 nm for each well using a multiwell spectrophotometer. The experiment was performed in triplicate.

### 2.9. Lactate Dehydrogenase Assay

LDH activity was detected using a LDH activity assay kit according to the manufacturer's instructions [24]. Absorbance values were measured at 490 nm with a reference wavelength of 655 nm, using a 96-well microplate reader (Tecan Systems Inc., Oberdiessbach, Switzerland). The experiment was repeated at least three times.

### 2.10. Reactive Oxygen Species Measurement

The amount of intracellular ROS was measured by the change of fluorescence resulting from oxidation of DCFH-DA, a membrane-permeable fluorescent probe [23]. After treated with SCM-198 for 2 h and OGD/R injury, cells were washed in PBS and incubated with 10 *μ*M of DCFH-DA for 30 min at 37°C in the dark. The cells were washed with serum-free DMEM to remove the free molecules of the dye. The fluorescence of intracellular ROS was detected by both fluorescence microscopy (Zeiss, Oberkochen, Germany) and microplate reader (TecanSystems Inc., Oberdiessbach, Switzerland).

### 2.11. Measurement of BBB Permeability In Vitro

Then we tested the putative function of SCM-198 in regulating the in vitro BBB permeability as previously described [25]. The bEnd.3 cells were seeded on 24-well transwell polycarbonate insert filters (diameter: 24 mm; pore size: 3 *μ*m; BD, USA) at a confluent density of 2 × 10^5^ cells/cm^2^ and incubated for 2 days. At 1 h before the end of the reoxygen period, solutions of FITC-labeled dextran solution were added to the apical chamber. The fluorescence intensity of FITC-dextran was detected at the excitation wavelength of 485 nm and the emission wavelength of 520 nm with a microplate reader (Tecan Systems Inc., Oberdiessbach, Switzerland). All samples were performed in triplicate.

### 2.12. Lentivirus Production and Transduction

Lentiviral plasmids and packaging plasmids were cotransfected into HEK293 cells to generate lentivirus by lipofectamine 2000 (Life Technologies) according to the manufacturer's instructions. The recombinant virus-containing medium was collected 72 h after transfection, and then the media was centrifuged at 5000*g* for 30 min to remove cell debris; at last, the supernatants were concentrated by PEG-it virus precipitation solution (SBI, USA) to obtain virus particles. After the bEnd.3 cells reached 60–70% confluence, the abovementioned lentiviral particles were used to transducer at a multiplicity of infection (MOI) of 50. The cells were used for the next experiment until at least 80%. The overexpression of adenovirus of HDAC4 was obtained from Gene Pharma (Shanghai, China). The efficiency of transduction was evaluated by the rate of GFP-positive cell number counted under a fluorescent microscope (Leica, Wetzlar, Germany).

### 2.13. Western Blot

Western blot analyses were performed as previously described [11]. The frozen tissues were cut into small pieces and homogenized in RIPA buffer and then centrifuged at 12,000*g* for 10 min at 4°C to separate soluble from insoluble fractions. Equal amounts of proteins for each group mixed with loading buffer were separated by 10% SDS-polyacrylamine gel and transferred to nitrocellulose filter membranes.

Each membrane was incubated with specific antibodies as follows: MMP-9, ZO-1, occludin, claudin-5, HDAC4, NOX4, and GAPDH. Immunoreactive proteins were visualized using the ECL western blotting detection kit (Thermo Fisher Scientific Inc., Boston, USA), according to the manufacturer's instructions. To measure the expression of each protein, the relative intensity was calculated by comparing with the intensity of GAPDH using densitometry (Bio-Rad, USA).

### 2.14. Real-Time RT-PCR

The total RNA was isolated from the brain cortex and striatum using TRIzol Reagent (Takara, Japan) as described in the manufacturer's instructions. After quantifying the amount of extracted total RNA, 500 ng template RNA was reversely transcribed to single-stranded cDNA with a Prime Script 1st strand cDNA synthesis kit (Takara, Japan) according to the manufacturer. Real-time PCR was performed on a BIO-RA- D IQ5 system (Bio-Rad, USA). The relative differences in gene expression between groups were expressed using cycle time (Ct) values. Briefly, the Ct values of the interested genes were first normalized with GAPDH of the same sample and then the relative differences between control and treatment groups. The primer sequences that were used in the real-time PCR analyses were provided in Supplementary Table 1 available online at https://doi.org/10.1155/2017/7150376.

### 2.15. Immunofluorescent Staining

Immunofluorescence was assessed as described earlier [26]. Following reoxygenation for 4 h, cells were washed with PBS and fixed in 4% paraformaldehyde for 30 min at room temperature, then washed with PBS 3 times. After permeabilizing with 0.1% Triton X-100, cells were blocked with 3% BSA for 30 min. Subsequently, the cells were incubated at 4°C overnight with the primary antibodies as follows: ZO-1 and occludin. After washing off the primary antibodies, the cells were incubated with Alexa Fluor 488- or 568-conjugated secondary antibodies (1 : 500, Thermo Fisher Scientific) at room temperature for 1 h. Following three times of PBS washes, the cell nuclei were stained using 4′,6-diamidino-2-phenylindole (DAPI, Beyotime).

To stain the F-actin stress fibres, cells were exposed to rhodamine phalloidin (1 : 50, Cytoskeleton) for 20 min, DAPI for staining the nuclei. Fluorescence staining was viewed with a laser scanning confocal microscope (Zeiss, Oberkochen, Germany).

### 2.16. Statistical Analysis

GraphPad Prism 5.0 software was used for analysis. Every two groups were compared by 1-way ANOVA with Tukey's post hoc test for *P* values. The Mann–Whitney *U* test was used for the statistical analysis of neurological deficits. All values are expressed as mean ± SEM. Values of *p* < 0.05 were considered to state statistical significance.

## 3. Results

### 3.1. SCM-198 Ameliorated Ischemia-Reperfusion Injury

**Figure 1:**
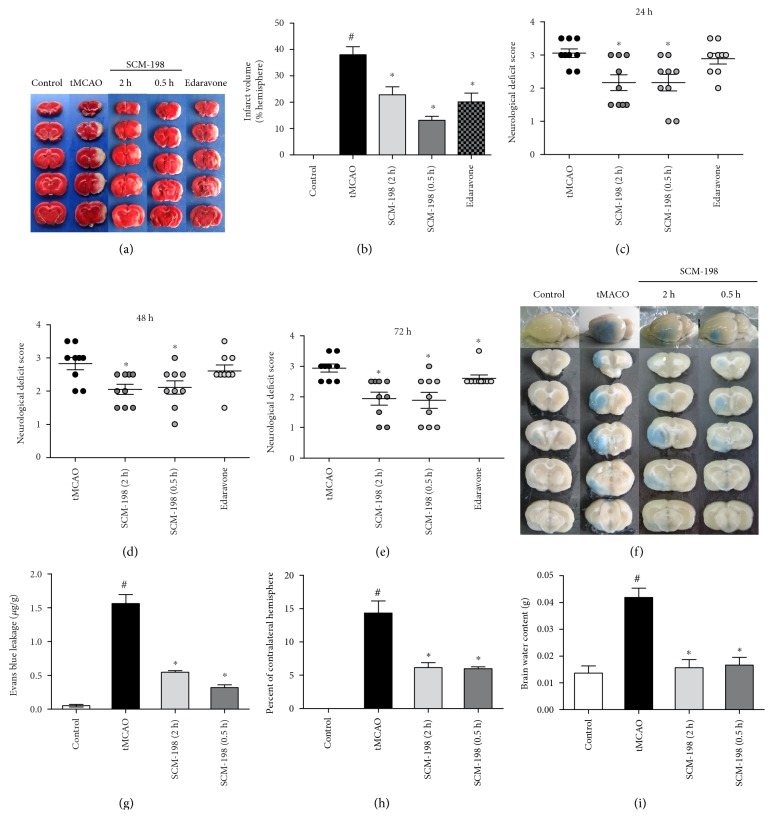
Posttreatment with SCM-198 significantly protected against the damage induced by tMCAO. The rats were subjected to 90 min MCAO before reperfusion. SCM-198 decreased the infarct volume and improved neurological scores. (a) Representative pictures of coronal sections from ischemic rat brain stained with TTC. (b) The quantitative analysis of the number of infarct size. *F* (4, 31) = 35.04, *P* < 0.0001. (c–e) Neurological function was ameliorated by SCM-198 after ischemia reperfusion. Values are expressed as mean ± SEM. ^#^*p* < 0.05 versus the control group, ^∗^*p* < 0.05 versus the tMCAO group, *n* = 8. Posttreatment with SCM-198 maintained BBB integrity. (f, g) At the end of treatment, the Evans blue dye was injected into the vein following 24 h circulation. (f) Representative pictures of coronal sections from rat brain stained with Evans blue dye after ischemic reperfusion. (g) The quantitative analysis of Evans blue leakage after ischemia reperfusion, *F* (3, 39) = 59.33, *P* < 0.0001. Edema volume ((h), *F* (3, 20) = 29.14, *P* < 0.0001) and water content ((i), *F* (3, 52) = 18.06, *P* < 0.0001) were decreased by SCM-198 after ischemia reperfusion. Values are expressed as mean ± SEM. ^#^*p* < 0.05 versus the control group, ^∗^*p* < 0.05 versus the tMCAO group, *n* = 6.

To test the therapeutical effect of SCM-198 on I/R, rats were subjected to 90 min of ischemia followed by 72 h of reperfusion. We set three time points to determine the therapeutic window: treatment at 0.5, 2, and 6 h postreperfusion. The brain infarct size was examined by TTC staining (Figure 1(a)), and we found that I/R markedly increased the infarct volume approximately up to 38.00 ± 8.10%, while postinjection of SCM-198 could significantly decrease the infarct volume (*F* (4, 31) = 35.04, *P* < 0.0001, Figure 1() (b)), notably at 0.5 h after reperfusion which was 13.13 ± 4.42%. SCM-198 revealed better protective effect against infarct volume than Edaravone which decreased the infarct volume only to 20.14 ± 8.86%. As neurological deficit caused by ischemia stroke is another big consequence, we observed and scored the behavior at 24, 48, and 72 h postsurgery. Compared with the tMCAO group, the neurological scores of treatment with SCM-198 and edaravone were significantly decreased at corresponding day (Figures 1(c)–1(e)). Moreover, we explored that treatment with SCM-198 at 0.5 h after surgery had the best effect. We also examined 6 h treatment after I/R, but there was no evident effect (data not shown). From the above results, we could safely conclude that SCM-198 improved neurological deficit, as well as decreased infarct volume.

### 3.2. The Effect of SCM-198 on BBB Breakdown

We know that stroke develops various damages to BBB integrity leading to the swell of brain tissue. Therefore, protection of BBB function can be a crucial aspect for reducing ischemic injury. To confirm the further mechanism of SCM-198 on stroke, we decided to focus on the protective effect against BBB breakdown. EB dye is always used as a marker of albumin effluxion to evaluate BBB permeability. EB can easily permeate the BBB after brain insult by I/R. We used EB to detect whether SCM-198 could maintain the intact BBB. Representative photos of EB dye in rat brain tissues are shown in Figure 1(f). We compared EB leakage in the ipsilateral hemisphere among all groups. SCM-198 could drastically reduce EB leakage when compared with the tMCAO group (Figure 1(f)). Treatment with SCM-198 at 2 h and 0.5 h after ischemic stroke markedly diminished EB leakage (Figure 1(f)). The quantitative analysis of EB leakage after I/R is shown in Figure 1(g) (*F* (3, 39) = 59.33, *P* < 0.0001). Correspondingly, SCM-198 could decrease the brain edema volume (*F* (3, 20) = 29.14, *P* < 0.0001, Figure 1(h)) and water content in the ipsilateral hemisphere subjected with I/R injury (*F* (3, 52) = 18.06, *P* < 0.0001, Figure 1(i)). These results suggested that SCM-198 may offer the protection against onset of BBB leakage.

### 3.3. SCM-198 Decreased the Degradation of Tight Junctions in Ischemic Brain

**Figure 2:**
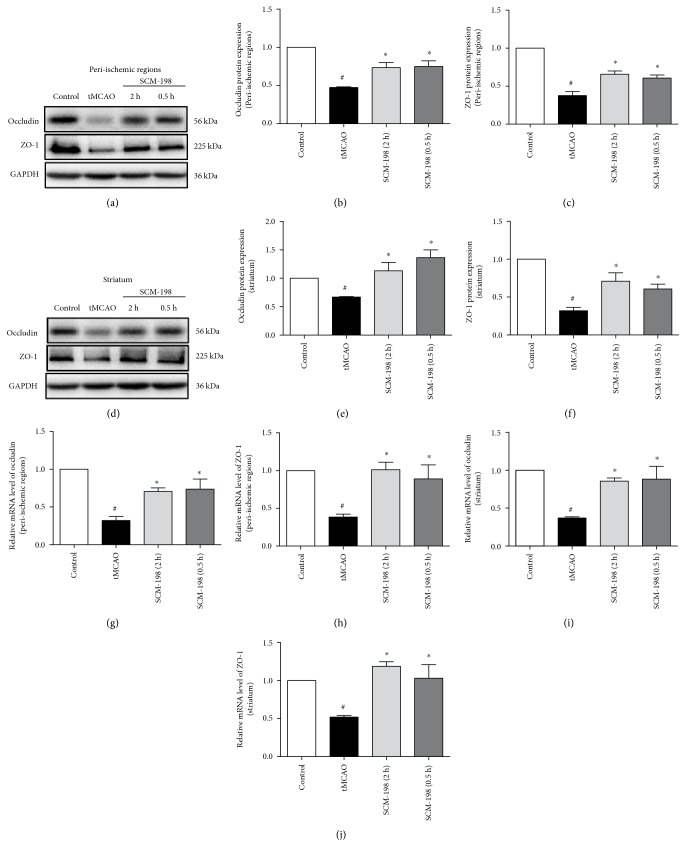
SCM-198 protected against TJ degradation induced by cerebral ischemic reperfusion. (a, d) Western blots of peri-ischemic region and striatum tissues in the tMCAO model showed increased the loss of occludin and ZO-1 levels, but reversed by SCM-198 treatment. GAPDH was used as the loading control. (b-c, e-f) The quantitative analysis occludin and ZO-1 levels were calculated and expressed relative to control. *F* (3, 12) = 18.65, *P* < 0.0001, Figure 2(b); *F* (3, 22) = 38.22, *P* < 0.0001, Figure 2(c); *F* (3, 8) = 8.228, *P* = 0.0079, Figure 2(e); *F* (3, 16) = 17.23, *P* < 0.0001, Figure 2(f). (g–j) Posttreatment with SCM-198 at 2 h and 0.5 h after reperfusion notably improved occludin and ZO-1 mRNA expression. SCM-198 revealed significantly protection. *F* (3, 6) = 25.11, *P* = 0.0009, Figure 2(g); *F* (3, 8) = 7.631, *P* = 0.0099, Figure 2(h); *F* (3, 8) = 10.04, *P* = 0.0044, Figure 2(i); *F* (3, 8) = 9.389, *P* = 0.0053, Figure 2(j). Values are expressed as mean ± SEM. ^#^*p* < 0.05 versus the control group, ^∗^*p* < 0.05 versus the tMCAO group, *n* = 4.

As we know, the cortex and striatum are the most sensitive to reperfusion, so we divided the brain into peri-ischemic region in the cortex and striatum. I/R causes the degradation of TJs, such as ZO-1 and occludin, which results in the loss of BBB function [27]. We examined the mRNA and protein levels of ZO-1 and occludin in the peri-ischemic region and striatum of rat brains. Their protein and mRNA levels, in both peri-ischemic region and striatum, were sharply decreased after I/R injury, while SCM-198 could dramatically restore the degradation (Figure 2(a); *F* (3, 12) = 18.65, *P* < 0.0001, Figure 2(b); *F* (3, 22) = 38.22, *P* < 0.0001, Figure 2(c); Figure 2(d); *F* (3, 8) = 8.228, *P* = 0.0079, Figure 2(e); *F* (3, 16) = 17.23, *P* < 0.0001, Figure 2(f); *F* (3, 6) = 25.11, *P* = 0.0009, Figure 2(g); *F* (3, 8) = 7.631, *P* = 0.0099, Figure 2(h); *F* (3, 8) = 10.04, *P* = 0.0044, Figure 2(i); *F* (3, 8) = 9.389, *P* = 0.0053, Figure 2(j)).

**Figure 3:**
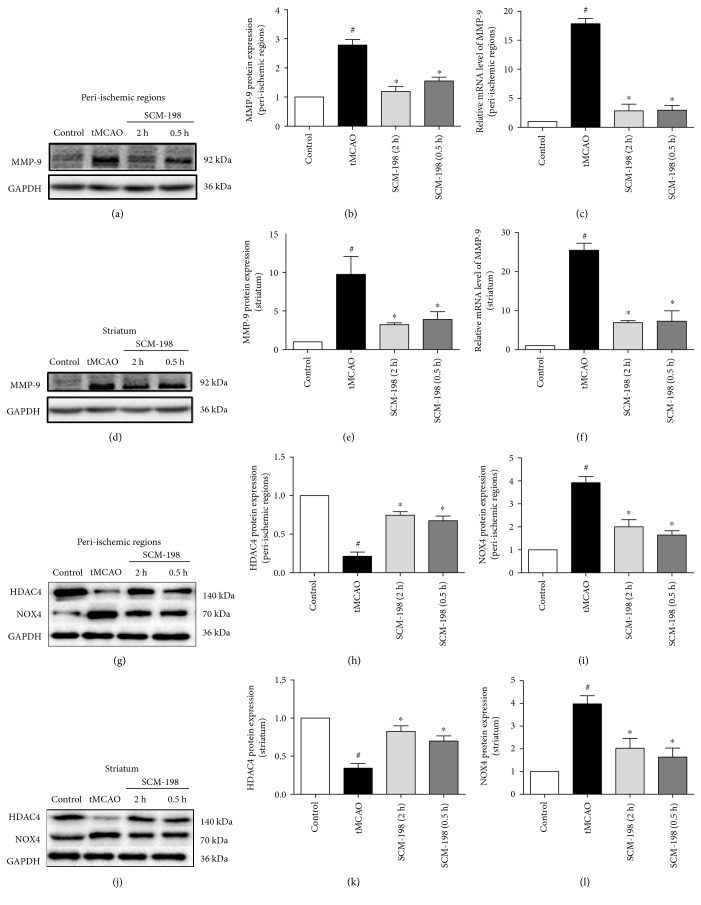
SCM-198 mediated the expression of MMP-9, NOX4, and HDAC4 in tMCAO rat. Reperfusion increased the loss of HDAC4 and the expression of MMP-9 and NOX4 in both peri-ischemic region and striatum tissues of the brain. (a–c) Reperfusion exacerbated the protein and mRNA expression of MMP-9 in peri-ischemic regions. SCM-198 significantly inhibited MMP-9 mRNA expression in the cortex. *F* (3, 9) = 38.73, *P* < 0.0001, Figure 3(b); *F* (3, 8) = 85.85, *P* < 0.0001, Figure 3(c). (d–f) Reperfusion improved the protein and mRNA expression of MMP-9 in the striatum. SCM-198 inhibited MMP-9 mRNA expression in the striatum. *F* (3, 10) = 14.65, *P* = 0.0005, Figure 3(e); *F* (3, 8) = 42.11, *P* < 0.0001, Figure 3(f); (g–l) Reperfusion increased the loss of HDAC4 and the expression of NOX4 in both peri-ischemic region and striatum tissues of the brain. GAPDH was used as the loading control. *F* (3, 15) = 51.79, *P* < 0.0001, Figure 3(h); *F* (3, 8) = 31, *P* < 0.0001, Figure 3(i); *F* (3, 11) = 24.47, *P* < 0.0001, Figure 3(k); *F* (3, 8) = 14.09, *P* = 0.0015, Figure 3(l). Values are expressed as mean ± SEM. ^#^*p* < 0.05 versus the control group, ^∗^*p* < 0.05 versus the tMCAO group, *n* = 4.

The TJs are reportedly degraded in I/R mainly by MMP-9 [28, 29] which is the predominant protease involving in BBB disruption following ischemic stroke [30]. It is also reported that MMP-9 is upregulated after ischemia and influences the delayed BBB opening which contributes to the irreversible increase in BBB permeability. So we speculated that SCM-198 may offer a protection through inhibiting the expression of MMP-9. The results confirmed our conjecture, western blot (Figure 3(a); *F* (3, 9) = 38.73, *P* < 0.0001, Figure 3(b); Figure 3(d); *F* (3, 10) = 14.65, *P* = 0.0005, Figure 3(e)), and real-time RT-PCR assays (*F* (3, 8) = 85.85, *P* < 0.0001, Figure 3(c), *F* (3, 8) = 42.11, *P* < 0.0001, Figure 3(f)) revealed that MMP-9 levels were evoked in the peri-ischemic region and striatum in tMCAO group, and SCM-198 treatment after surgery could remarkably reduce the expressions of MMP-9.

NOX4 and HDAC4 involve in mediating BBB permeability, and their expressions are changed by I/R [2, 31]. To determine whether SCM-198 affected expressions of NOX4 and HDAC4, the protein levels were tested in the peri-ischemic region and striatum.  Figure 3 (Figure 3(g); *F* (3, 15) = 51.79, *P* < 0.0001, Figure 3(h); *F* (3, 8) = 31, *P* < 0.0001, Figure 3(i); Figure 3(j); *F* (3, 11) = 24.47, *P* < 0.0001, Figure 3(k); *F* (3, 8) = 14.09, *P* = 0.0015, Figure 3(l)) suggested that I/R caused loss of HDAC4 but increase of NOX4 in both regions of the brain; treatment with SCM-198 enhanced the protein level of HDAC4 and inhibited the expression of NOX4. These results indicated that SCM-198 had a pivotal role in supporting BBB maintenance in vivo.

In order to confirm the protective action of SCM-198 against BBB breakdown and find out how SCM-198 exerted its effect, we used the OGD/R cell model for the further study.

### 3.4. SCM-198 Provided Protective Effect against OGD/R Insult

**Figure 4:**
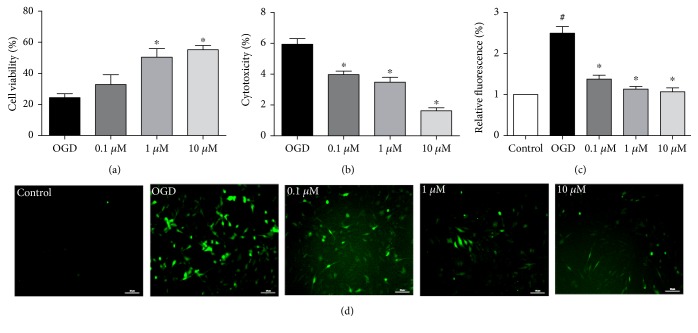
SCM-198 protected against ischemia-like injury in the in vitro BBB model. bEnd.3 cell line was exposed to 6 h of OGD followed by 4 h reperfusion after treated with different concentrations of SCM-198. (a) OGD/R markedly increased cell death, SCM-198 dose-dependently improved the cell viability, *F* (3, 14) = 12.57, *P* = 0.0003. (b) SCM-198 reduced the LDH leakage in the supernatant after OGD/R, *F* (3, 4) = 39.25, *P* = 0.002. (c) The fluorescence intensity of intracellular ROS, *F* (4, 12) = 43.35, *P* < 0.0001. (d) OGD/R produced abundant ROS in the cells, while SCM-198 significantly reduced the ROS formation, scale bar = 10 *μ*m. Values are expressed as mean ± SEM. ^#^*p* < 0.05 versus the control group, ^∗^*p* < 0.05 versus the OGD group, *n* = 3.

To clarify the mechanisms of the effect of SCM-198 on BBB disruption, we took an in vitro BBB model on bEnd.3 cell line followed OGD for 6 h and reoxygenation for 4 h. Meantime, we examined the expression of HIF-*α* to confirm the success of this model (Supplementary Figure 1). The cell viability was determined 4 h after reoxygenation by the CCK8 method. Results revealed that cell death in OGD/R group was significantly increased compared with the control group (*F* (3, 14) = 12.57, *P* = 0.0003, Figure 4(a)). Pretreatment with different concentrations of SCM-198 had a dose-dependent protective effect, especially the 10 *μ*M group which could increase the cell viability from 24.49 ± 5.84% to 55.24 ± 5.95%. At the same time, LDH assay was used for assessing cytotoxicity. We found that OGD/R under our condition increased LDH leakage into the cell culture media compared with the control group (*p* < 0.05). And SCM-198 treatment could markedly decrease the LDH release (*F* (3, 4) = 39.25, *P* = 0.002, Figure 4(b)). These results indicated that SCM-198 provided protection against OGD/R injury.

### 3.5. SCM-198 Decreased ROS Production in bEnd.3 Cell Line

We detected OGD/R-induced ROS production by DCF-DA reagent, a fluorescent probe used for visualizing ROS. OGD/R enriched DCF-DA-positive cells compared with the control group (*p* < 0.05). SCM-198 notably cut ROS generation and reduced the positive cell number in a concentration-dependent manner compared with the OGD/R group. There was no apparent difference between the SCM-198 treatment group and control group (Figure 4(d)). We also calculated the fluorescent intensity by microplate reader. The graph showed that SCM-198 significantly decreased the fluorescent intensity compared with the OGD/R group (*F* (4, 12) = 43.35, *P* < 0.0001, Figure 4(c)).

### 3.6. SCM-198 Maintained the BBB Integrity In Vitro

**Figure 5:**
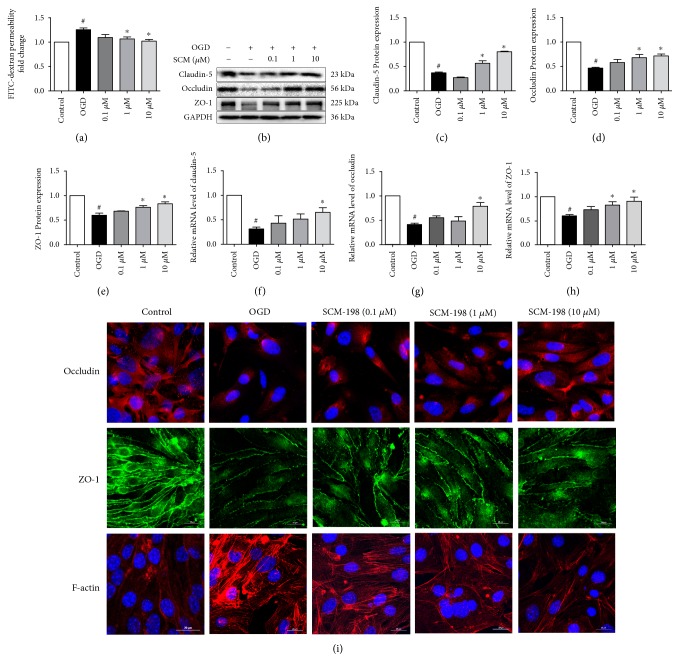
SCM-198 reduced the degradation of TJs induced by OGD/R. (a) FITC-dextran was used to determine BBB integrity. After reperfusion the leakage of dextran in the plate was remarkably increased. SCM-198 could reduce the leakage, *F* (4, 10) = 6.671, *P* = 0.007. (b) Expressions of TJs, occludin, claudin-5, and ZO-1 were evaluated by western blot 4 h after reperfusion. OGD/R induced the obvious loss of TJs, and SCM-198 reversed this loss. (c–e) The results were quantified and expressed relative to control, *F* (4, 10) = 133.1, *P* < 0.0001, Figure 5(c); *F* (4, 14) = 26.32, *P* < 0.0001, Figure 5(d); *F* (4, 14) = 22.99, *P* < 0.0001, Figure 5(e). (f–h) The expressions of mRNA levels were detected by real time RT-PCR. SCM-198 could reduce the degradation of TJs at the level of mRNA, *F* (4, 10) = 7.764, *P* = 0.0041, Figure 5(f); *F* (4, 15) = 5.983, *P* = 0.0044, Figure 5(g); *F* (4, 21) = 12.70, *P* < 0.0001, Figure 5(h). (i) Immunofluorescence analysis of occludin and ZO-1, and rhodamine-conjugated phalloidin for stress fibre. Scale bar = 20 *μ*m. The expressions of occludin and ZO-1 were significantly reduced by OGD/R while SCM-198 prevented their reduction. SCM-198 also decreased the formation of stress fibre. Values are expressed as mean ± SEM. ^#^*p* < 0.05 versus the control group, ^∗^*p* < 0.05 versus the OGD group, *n* = 3.

FITC-dextran is always used as a fluorescent tracer for evaluating BBB TJ function. Under physiological conditions, due to BBB, the endothelial membrane is impermeable for macromolecules to cross the barrier. But when the cell is subjected to OGD/R injury, FITC-dextran flux observably increased, which indicated disruption of the barrier. SCM-198 reduced the flux of FITC-dextran, suggesting SCM-198 could maintain the integrity of BBB in vitro (*F* (4, 10) = 6.671, *P* = 0.007, Figure 5(a)).

Western blots showed that TJ proteins, claudin-5, occludin, and ZO-1 were dramatically degraded in the OGD/R group. This reduction of TJs may interpret the vast increase in barrier permeability. But SCM-198 could signally reverse the degradation of TJs (Figure 5(b); *F* (4, 10) = 133.1, *P* < 0.0001, Figure 5(c); *F* (4, 14) = 26.32, *P* < 0.0001, Figure 5(d); *F* (4, 14) = 22.99, *P* < 0.0001, Figure 5(e)). The results were confirmed by real-time RT-PCR, and the mRNA expressions of claudin-5, occludin, and ZO-1 were markedly reduced at 4 h post-OGD/R. SCM-198 prevented the reduction of these three proteins (*F* (4, 10) = 7.764, *P* = 0.0041, Figure 5(f); *F* (4, 15) = 5.983, *P* = 0.0044, Figure 5(g); *F* (4, 21) = 12.70, *P* < 0.0001, Figure 5(h)). Immunofluorescence analysis also showed that OGD/R weakened occludin and ZO-1 expression when compared with intact ZO-1 and occludin in the control group. But the loss of ZO-1 and occludin was recovered after treatment with SCM-198 (Figure 5(i)). According to the above findings, we also found abundant linear stress fibre formation, which accounts for cell contraction and hyperpermeability [32–35], which was stained by rhodamine dye after OGD/R (Figure 5(i)).

These data manifested that OGD/R touched off robust loss of occludin, claudin-5, and ZO-1 while SCM-198 could improve the expressions. These results were consistent with the findings in vivo.

### 3.7. SCM-198 Ameliorated BBB Injury via Enhancing HDAC4

**Figure 6:**
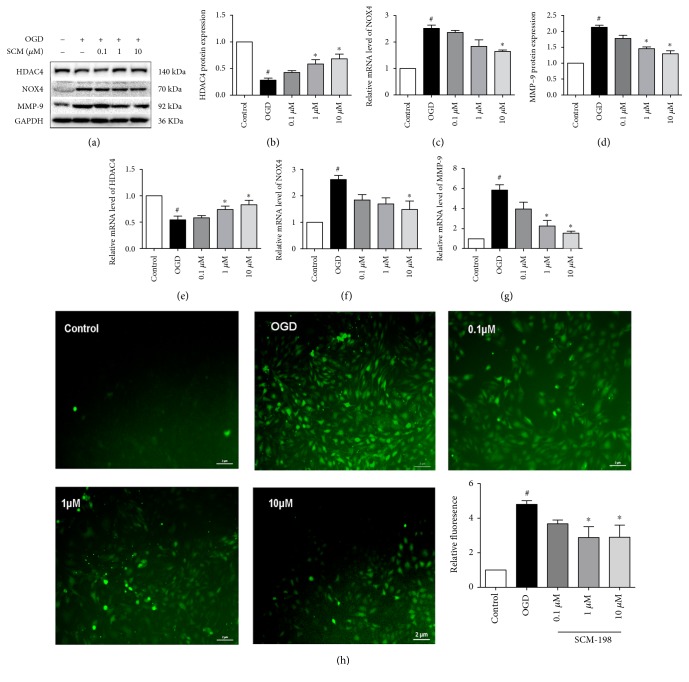
SCM-198 mediated the expression of MMP-9, NOX4, and HDAC4 in vitro. (a–d) Western blot images and quantitative analysis of MMP-9, NOX4, and HDAC4 were shown, *F* (4, 12) = 20.46, *P* < 0.0001, Figure 6(b); *F* (4, 19) = 20.6, *P* < 0.0001, Figure 6(c); *F* (4, 10) = 34.23, *P* < 0.0001, Figure 6(d). (e–g) The mRNA levels of HDAC4, NOX4, and MMP-9 were estimated by real-time RT-PCR, *F* (4, 10) = 9.474, *P* = 0.002, Figure 6(e); *F* (4, 10) = 7.831, *P* = 0.004, Figure 6(f); *F* (4, 10) = 39.81, *P* < 0.0001, Figure 6(g). (h) The HDAC4 inhibitor Taq (0.2 *μ*M) or vehicle was applied 1 h before until the end of the experiment. Taq exacerbated the production of ROS, while SCM-198, 1 and 10 *μ*M, could still reduce the ROS formation, *F* (4, 14) = 9.319, *P* = 0.0007, scale bar = 2 *μ*m. Values are expressed as mean ± SEM. ^#^*p* < 0.05 versus the control group, ^∗^*p* < 0.05 versus the OGD group, *n* = 3.

To identify whether the signals causing BBB damage in vivo also occur in cell model in vitro, we measured the protein and mRNA levels of HDAC4, NOX4, and MMP-9. In the OGD/R group, both protein and mRNA levels of HDAC4 were notably reduced (Figures 6(a), 6(b), and 6(e)), while NOX4 and MMP-9 were significantly increased after reoxygenation (Figures 6(a), 6(c), 6(), 6(f), and 6(g)). Consistent with the results in vivo, SCM-198 could exert the protection by enhancing the expression of HDAC4 and inhibiting the expression of NOX4 and MMP-9 (Figure 6(a); *F* (4, 12) = 20.46, *P* < 0.0001, Figure 6(b); *F* (4, 19) = 20.6, *P* < 0.0001, Figure 6(c); *F* (4, 10) = 34.23, *P* < 0.0001, Figure 6(d); *F* (4, 10) = 9.474, *P* = 0.002, Figure 6(e); *F* (4, 10) = 7.831, *P* = 0.004, Figure 6(f); *F* (4, 10) = 39.81, *P* < 0.0001, Figure 6(g)). These results suggested that SCM-198 could also exhibit protective effect on BBB integrity in vitro by regulating the expression of HDAC4, NOX4, and MMP-9.

HDAC4 and NOX4 are two proteins which exist in the same signaling pathway, but the regulative relationship between HDAC4 and NOX4 is not clear. To explored whether HDAC4 was responsible for the expression of NOX4, we used adenovirus or specific inhibitor Taq to overexpress or inhibit HDAC4 in bEnd.3 cells and then exposed these cells to OGD/R. Western blot results revealed that overexpression of HDAC4 by adenovirus inhibited the increase of NOX4 (Supplementary Figure 2A-B), while Taq further increased the expression of NOX4 at 0.2 *μ*M (Supplementary Figure 2C). On the other hand, overexpression of NOX4 by lentivirus or inhibition of NOX4 with DPI and GKT137831 could not influence the expression of HDAC4 (Supplementary 3). Therefore, we speculated that HDAC4 could inhibit the expression of NOX4 under our OGD/R condition.

**Figure 7:**
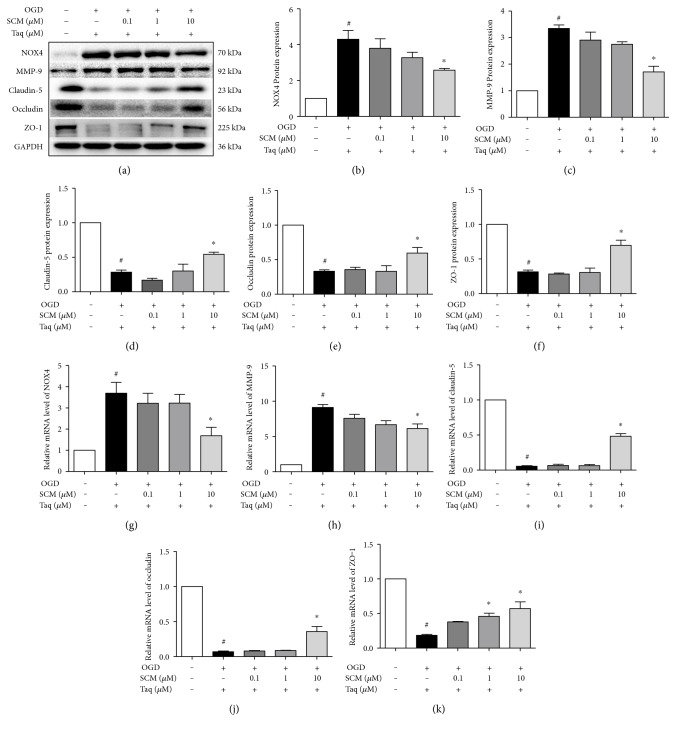
SCM-198 maintained the BBB integrity via enhancing the expression of HDAC4. The HDAC4 inhibitor Taq (0.2 *μ*M) or vehicle was applied 1 h before until the end of the experiment. (a–f) Western blot images and quantitative analysis suggested inhibition of HDAC4 increased the expression of NOX4 and MMP-9, and treatment with 10 *μ*M SCM-198 reduced the raised levels of NOX4 and MMP-9. Meanwhile, SCM-198 decreased the loss of TJs. The results revealed SCM-198 could protect against the degradation of TJs via improving the expression of HDAC4, *F* (4, 15) = 13.24, *P* < 0.0001, Figure 7(b); *F* (4, 13) = 28.16, *P* < 0.0001, Figure 7(c); *F* (4, 10) = 43.70, *P* < 0.0001, Figure 7(d); *F* (4, 10) = 27.45, *P* < 0.0001, Figure 7(e); *F* (4, 10) = 50.05, *P* < 0.0001, Figure 7(f); (g–k) The mRNA levels of NOX4, MMP-9, and TJs were estimated by real-time RT-PCR, *F* (4, 10) = 8.332, *P* = 0.0032, Figure 7(g); *F* (4, 10) = 39.14, *P* < 0.0001, Figure 7(h); *F* (4.10) = 401, *P* < 0.0001, Figure 7(i); *F* (4.10) = 144.9, *P* < 0.0001, Figure 7(j); *F* (4.10) = 39.03, *P* < 0.0001, Figure 7(k). Values are expressed as mean ± SEM. ^#^*p* < 0.05 versus the control group, ^∗^*p* < 0.05 versus the OGD group, *n* = 3.

Next, we examined whether HDAC4 was involved in the regulation of BBB integrity. The results showed that Taq enhanced the ROS formation (*F* (4, 14) = 9.319, *P* = 0.0007, Figure 6(h)) and increased the protein and mRNA levels of NOX4 and MMP-9 in bEnd.3 cells (Figure 7(a), *F* (4, 15) = 13.24, *P* < 0.0001, Figure 7(b); *F* (4, 13) = 28.16, *P* < 0.0001, Figure 7(c); *F* (4, 10) = 43.70, *P* < 0.0001, Figure 7(d); *F* (4, 10) = 27.45, *P* < 0.0001, Figure 7(e); *F* (4, 10) = 50.05, *P* < 0.0001, Figure 7(f); *F* (4, 10) = 8.332, *P* = 0.0032, Figure 7(g); *F* (4, 10) = 39.14, *P* < 0.0001, Figure 7(h)). Furthermore, Taq abolished the protection of HDAC4 on BBB integrity by decreasing the expression of TJ protein and mRNA such as claudin-5, occludin, and ZO-1 (Figures 7(a) and 7(d)–7(f), *F* (4.10) = 401, *P* < 0.0001, Figure 7(i); *F* (4.10) = 144.9, *P* < 0.0001, Figure 7(j); *F* (4.10) = 39.03, *P* < 0.0001, Figure 7(k)). Treatment with SCM-198 reduced the ROS production (Figure 6(h)) and the overexpression of NOX4 and MMP-9 induced by Taq (Figures 7(a)–7(c) and 7(g)-7(h)). Similarly, treatment with SCM-198 increased the mRNA and protein levels of these TJ proteins (Figures 7(a), 7(d)–7(f), and 7(i)–7(k)). Together these results indicated that SCM-198 may exert the protection effect on BBB through enhancing the expression of HDAC4 which could regulate NOX4 and MMP-9.

## 4. Discussion

In this article, we used classic tMCAO rat model in vivo and reperfusion post-OGD cell model in vitro to explore the potential of SCM-198 as a therapeutic approach for reperfusion-induced BBB disrupture. We found that SCM-198 significantly decreased infarct volume and ameliorated neurological deficit in the tMCAO model. Moreover, SCM-198 could also reduce the cell injury caused by OGD/R in vitro. Further study about the mechanism demonstrated that HDAC4 could inhibit the expression of NOX4 and MMP-9 and then improve TJ levels, therefore protect against BBB breakdown. In conclusion, we first revealed that HDAC4 was involved in regulating BBB integrity and SCM-198 had the protective effects against BBB leakage through enhancing the expression of HDAC4.

The brain suffers from ischemia-induced vast loss of oxygen and nutrient leading to tissue damage, especially the cortex and striatum regions [36], and reperfusion exacerbates this insult because of the fresh oxygen [1]. We found that both cortex and striatum regions in the tMCAO animals appeared plenty of infarct volume, approximately 40% of the whole brain (Figures 1(a) and 1(b)). SCM-198 and Edaravone could significantly reduce the infarct area and reduce neurological deficit scores (Figures 1(c) and 1(e)). Furthermore, treatment with SCM-198 at 0.5 h postsurgery revealed better therapeutic effect on infarct area and neurological deficit score than Edaravone (Figure 1).

BBB is a specialized structure between the brain tissue and blood circulation to maintain the homeostasis of microenvironment and avoid the harm from the exogenous compounds. Study showed that treatment with tPA often accompanies with lethal complication of brain edema due to reperfusion insult which contribute to the disrupture of BBB. In our study, I/R-induced BBB disruption was confirmed by EB dye which crossed from blood into parenchyma (Figure 1(f)). Furthermore, the increase of bEnd.3 cells permeability induced by OGD/R in the in vitro model was tested by FITC-dextrans. SCM-198 remarkably decreased EB and FITC-dextran leakage (Figure 5(a)). Besides, we also detected that I/R significantly increased the water content and edema volume of the ipsilateral brain compared with the control rats. SCM-198 decreased the brain edema volume and water content in the ipsilateral hemisphere (Figures 1(h) and 1(i)). These results indicated that SCM-198 played the protective effect against BBB breakdown both in vivo and in vitro.

Following stroke, BBB disrupture has two phases: the initial opening occurs within hours after stroke onset and the second phase comes 24–48 h later. MMPs, especially MMP-2 and MMP-9, are involved in such early and late phases. The early BBB breakdown is mainly caused by MMP-2 which increased in the early phase of the tMCAO model [37]. But in our condition, the mRNA expression of MMP2 was unchanged between each group (Supplementary Figure 4). Correspondingly, MMP-9 was elevated during the delayed barrier disrupture, from 4 h to 4 days. The upregulation of MMP-9 leads to the production of stress fibre and entire degradation of TJs, in addition to complete disrupture of BBB and brain edema [38].

In the clinic, MMP-9 is mainly derived from infiltrating neutrophils and microvessel endothelium after ischemic stroke in humans [39]. MMP-9 is considered as the predominant protease which can break the integrity of BBB following ischemic stroke. We supposed that SCM-198 may protect the further lesion by regulating the expression of MMP-9. We examined the expression of MMP-9 and found that MMP-9 was indeed declined by SCM-198 treatment. While TJs, which could be regulated by MMP-9, play critical function in maintaining BBB integrity. In our study, we found SCM-198 could enhance the mRNA and protein levels of them and then defend the insult induced by I/R. These suggested that SCM-198 has a beneficial effect on BBB damage by inhibiting MMP-9 levels and improve TJ expression (Figures 2, 3, 5, and 6).

It is well known that nuclear factor kappa-B (NF-*κ*B) and reactive oxygen species (ROS) are involving of MMP-9 activation during ischemia [40]. ROS, including hydrogen peroxide, superoxide anion, and hydroxyl radical, are induced by ischemia following reperfusion which contributes to the critical injury to the brain tissue and BBB disruption [41–43]. When the brain is subjected to oxidative stress, NOXs are activated and then the electrons are transferred from NADPH to oxygen producing abundant ROS. NOX family is the primary source of ROS [44], which comprises seven members: NOX1, NOX2, NOX3, NOX4, NOX5, dual oxidase- (Duox-) 1, and Duox-2. Of these NOX isoforms identified hitherto [45–47], NOX1, NOX2, and NOX4 are expressed in the brain and involved in BBB dysfunction after cerebral ischemia and reperfusion. The recent research had proved that NOX4^−/−^ mice exhibited the decrease of infarct volume, oxidative stress, BBB disruption, and neuronal apoptosis in the tMCAO model. But the absent of NOX2 had no effect on infarct area, BBB leakage, neuronal apoptosis, or functional outcome after tMCAO. So the researchers concluded that deletion of NOX4, not NOX1 or NOX2, exerted the protective effect [48]. In our study, we found that the expression of NOX2 did not change in the tMCAO model (Supplementary Figure 5), suggesting that NOX2 did not play a predominant role in our situation. As we know, NOX4 is considered as the major source of ROS. NOX4, first discovered in the kidney, is mainly distributed in neuron and endothelial cells of the brain [1, 43]. Ischemic stroke induces NOX4 activated [48], and previous research revealed that inhibition of NOX4 could suppress the enhanced level of MMP-9 induced by tMCAO [47].

Based on these results, we believed that NOX4 may exert a main role in our tMCAO and OGD/R models. Our results showed that NOX4 expression was increased in both tMCAO and OGD/R models. Treatment with SCM-198 would reduce the expression of NOX4 and then protect against I/R and OGD/R injury (Figures 3 and 6). A number of transcription factors, for instance, NF-*κ*b[49], SMAD proteins [50], HIF1*α* [51], E2F [52], Nrf2 [53], Nrf3 [54] and STAT1/3 [55] have been shown to regulate NOX4 gene expression. However, few researchers focused on the epigenetic regulation of NOX4. Some studies confirmed that HDAC inhibition provided neuroprotection after cerebral ischemic insult or intracerebral hemorrhage [56–58]. Histone deacetylases (HDACs) are a protein family consisted of 18 proteins which could be categorized into 4 groups according to their structural and functional resemblance: class I (HADCs 1, 2, 3, and 8), class IIa (HDACs 4, 5, 7, and 9), class IIb (HDACs 6 and 10), class III (sirtuins 1–7), and class IV (HDAC 11) [59]. HDAC dysregulation is associated with brain disorders such as Huntington's disease, Alzheimer's disease, and ischemic stroke [60, 61]. Therefore, it could be a potential therapeutic target. He et al. examined the change of HDACs 1–11 induced by ischemia and reperfusion; the results suggested that only HDAC4/5 mRNA levels were significantly decreased while HDAC9 was prominently increased in ischemic hemisphere [31].

In our study, in both rat and cell model groups, the levels of HDAC4 were notably decreased, and treatment with SCM-198 could enhance the expression of HDAC4 (Figures 6(a) and 6(b)). Furthermore, we overexpressed or inhibited HDAC4 to clarify the regulative relationship between HDAC4 and NOX4. The results showed that HDAC4 could regulate the expression of NOX4 in the opposite direction. Therefore, we demonstrated that SCM-198 could regulate the expression of HDAC4 which modulated NOX4 level and then influenced the downstream of BBB disrupture (Figure 7).

## 5. Conclusion

**Figure 8:**
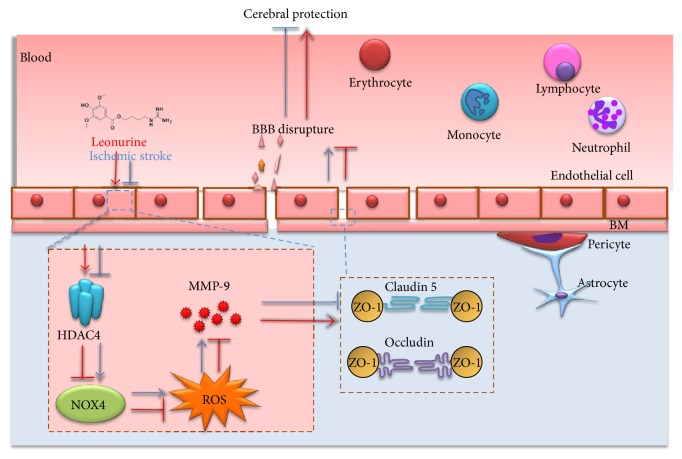
Schematic representation of the mechanisms of SCM-198 protection against ischemic stroke. SCM-198 protected BBB integrity by regulating the HDAC4/NOX4/MMP-9 tight junction pathway.

In summary, our research implied that SCM-198 reduced the infarct volume, ameliorated the neurological deficit, and protected against BBB leakage in vivo and in vitro. The underlying mechanism may be that SCM-198 could improve the level of HDAC4 which could regulate the expression of NOX4 and further influence the downstream pathway to exert the protective effect on BBB disrupture (Figure 8). SCM-198 may be a promising candidate for the treatment of cerebral ischemia and reperfusion.

## Supplementary Material

Supplementary Table 1: Real-time RT-PCR primer sequences. Supplementary Figure 1. The expression of HIF-α after OGD/R. The success of the in vitro model was confirmed by the expression of HIF-α. The level of HIF-α was increased at different time after reperfusion. Supplementary Figure 2. HDAC4 regulated the level of NOX4. A: The adenovirus overexpressed HDAC4 in normal condition. B: Overexpression of HDAC4 by adenovirus inhibited the increase of NOX4 induced by OGD/R. C: The specific inhibitor of HDAC4 named Tasquinimod further increased the expression of NOX4 especially at 0.2 μM. Supplementary Figure 3. NOX4 had no effect on the expression of HDAC4. A: The protein level of NOX4 was overexpressed by lentivirus in the normal cells. B: The expression of HDAC4 did not change after over-express of NOX4 under the normal condition. C, D: The inhibitors of NOX4, GKT 137831 and DPI, had no obvious impact on the expression of HDAC4 after OGD/R. Supplementary Figure 4. SCM-198 had no effect on MMP2. In our condition, the mRNA expression of MMP2 after 72h reperfusion had no significant change between control and tMCAO group. At the same time, SCM-198 had no effect on MMP2. Supplementary Figure 5. The expression of NOX2 in vivo. In our tMCAO model, the expression of NOX2 had no change.

## Abbreviations

tMCAO: Transient middle cerebral artery occlusion
BBB: Blood-brain barrier
OGD/R: Oxygen-glucose deprivation and reoxygenation
ROS: Reactive oxygen species
ZO: Zonula occludens
NOX: Nicotinamide adenine dinucleotide phosphate oxidases
MMPs: Matrix metalloproteinases
HDACs: Histone deacetylases
tPA: Tissue-type plasminogen activator
NVU: Neurovascular unit
TJs: Tight junctional proteins
Cav1: Caveolin-1
JAM: Junctional adhesion molecules
BMECs: Brain microvessel endothelium cells
NF-*κ*B: Nuclear factor kappa-B
CNS: Central nervous system
I/R: Ischemic reperfusion
CBF: Cerebral blood flow
TTC: 2,3,5-Triphenyltetrazolium chloride
BWC: Brain water content
LDH: Lactate dehydrogenase
EB: Evans blue
DPI: Diphenyleneiodonium
Taq: Tasquinimod.
