# Supplementary material for: Novel Therapeutic Effects of Leonurine On Ischemic Stroke: New Mechanisms of BBB Integrity
Source: Oxid Med Cell Longev. 2017 Jun 13;2017:7150376. doi: 10.1155/2017/7150376 (PMC5485366; doi:10.1155/2017/7150376)

**Supplementary Table 1 ：** Real-time RT-PCR primer sequences

**Rat**

| Gene | Forward (5’-3’) | Reverse (5’-3’) |
| --- | --- | --- |
| MMP9 | GACCTCAAGTGGCACCATCA | AGTCATCGATCACGTCTCGC |
| Occludin | GTGGCTTCCACACTTGCTTG | CTCCATAGCTACCGAAGCCG |
| ZO-1 | GTCCCTTACCTTTCGCCTGAA | CAGCTTGTGATACGTGCGAG |
| GAPDH | ATGTATCCGTTGTGGATCTGAC | CCTGCTTCACCACCTTCTTG |

**Mouse**

| Gene | Forward (5’-3’) | Reverse (5’-3’) |
| --- | --- | --- |
| HDAC4 | CATTGGAGGAGCTGCAGACA | GGAAGCCTGACGAACACTGA |
| NOX4 | AACACCTCTGCCTGCTCATT | GCTGCAGTTGAGGTTCAGGA |
| MMP9 | GTGGGACCATCATAACATCACA | CTCGCGGCAAGTCTTCAGAGTA |
| Occludin | GTGGCTTCCACACTTGCTTG | AAGCGATGAAGCAGAAGGCT |
| ZO-1 | CCACAAGGAGCCATTCCTGA | TAGGGTCACAGTGTGGCAAG |
| Claudin5 | GTTAAGGCACGGGTAGCACT | TACTTCTGTGACACCGGCAC |
| GAPDH | TTCAACGGCACAGTCAAGG | CGGCATGTCAGATCCACAA |

**Supplementary Fig. 1 The expression of HIF-α after OGD/R.** The success of the *in vitro* model was confirmed by the expression of HIF-α. The level of HIF-α was increased at different time after reperfusion


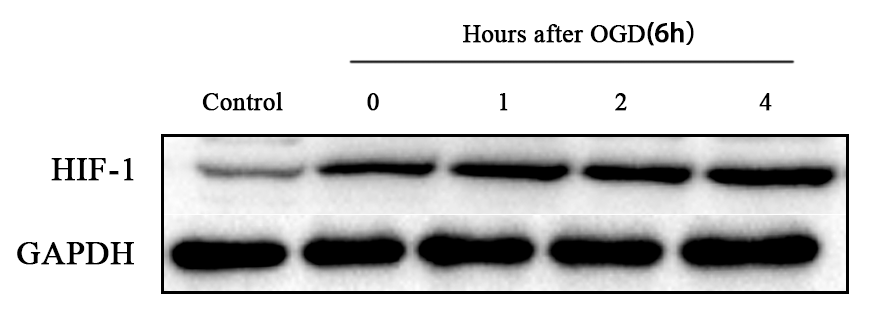


**Supplementary Fig. 2 HDAC4 regulated the level of NOX4.** A: The adenovirus overexpressed HDAC4 in normal condition. B: Overexpression of HDAC4 by adenovirus inhibited the increase of NOX4 induced by OGD/R. C: The specific inhibitor of HDAC4 named Tasquinimod further increased the expression of NOX4 especially at 0.2 μM.


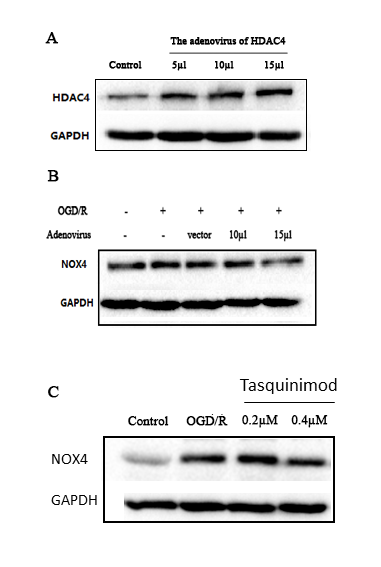


**Supplementary Fig. 3 NOX4 had no effect on the expression of HDAC4.** A: The protein level of NOX4 was overexpressed by lentivirus in the normal cells. B: The expression of HDAC4 did not change after over-express of NOX4 under the normal condition. C, D: The inhibitors of NOX4, GKT 137831 and DPI, had no obvious impact on the expression of HDAC4 after OGD/R.


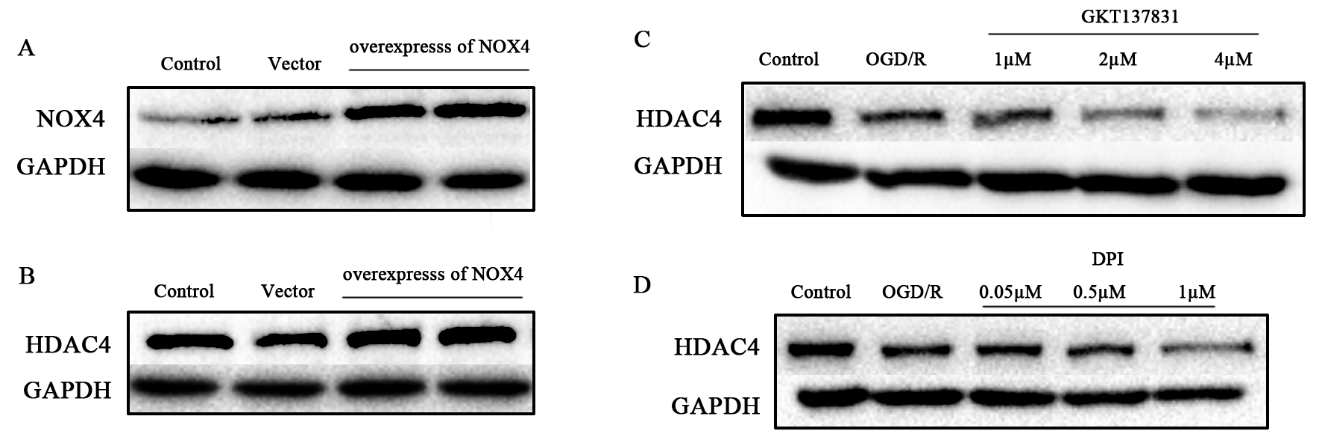


**Supplementary Fig. 4 SCM-198 had no effect on MMP2.** In our condition, the mRNA expression of MMP2 after 72h reperfusion had no significant change between control and tMCAO group. At the same time, SCM-198 had no effect on MMP2.

**
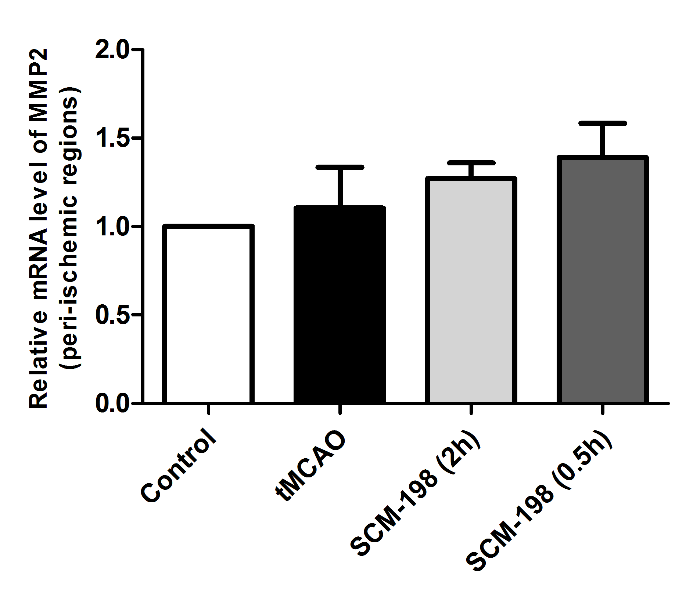
**

**Supplementary Fig. 5 The expression of NOX2 *in vivo*.** In our tMCAO model, the expression of NOX2 had no change.


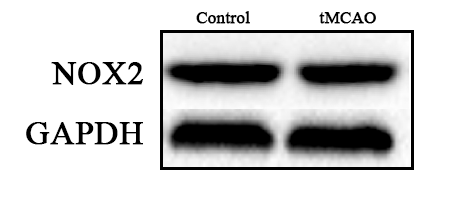

Supplement: Supplementary file 1 — Supplementary Table 1: Real-time RT-PCR primer sequences. Supplementary Figure 1. The expression of HIF-α after OGD/R. The success of the in vitro model was confirmed by the expression of HIF-α. The level of HIF-α was increased at different time after reperfusion. Supplementary Figure 2. HDAC4 regulated the level of NOX4. A: The adenovirus overexpressed HDAC4 in normal condition. B: Overexpression of HDAC4 by adenovirus inhibited the increase of NOX4 induced by OGD/R. C: The specific inhibitor of HDAC4 named Tasquinimod further increased the expression of NOX4 especially at 0.2 μM. Supplementary Figure 3. NOX4 had no effect on the expression of HDAC4. A: The protein level of NOX4 was overexpressed by lentivirus in the normal cells. B: The expression of HDAC4 did not change after over-express of NOX4 under the normal condition. C, D: The inhibitors of NOX4, GKT 137831 and DPI, had no obvious impact on the expression of HDAC4 after OGD/R. Supplementary Figure 4. SCM-198 had no effect on MMP2. In our condition, the mRNA expression of MMP2 after 72h reperfusion had no significant change between control and tMCAO group. At the same time, SCM-198 had no effect on MMP2. Supplementary Figure 5. The expression of NOX2 in vivo. In our tMCAO model, the expression of NOX2 had no change. [file 7150376.f1.docx]
